# External validation of the diffuse intrinsic pontine glioma survival prediction model: a collaborative report from the International DIPG Registry and the SIOPE DIPG Registry

**DOI:** 10.1007/s11060-017-2514-9

**Published:** 2017-05-30

**Authors:** Sophie E. M. Veldhuijzen van Zanten, Adam Lane, Martijn W. Heymans, Joshua Baugh, Brooklyn Chaney, Lindsey M. Hoffman, Renee Doughman, Marc H. A. Jansen, Esther Sanchez, William P. Vandertop, Gertjan J. L. Kaspers, Dannis G. van Vuurden, Maryam Fouladi, Blaise V. Jones, James Leach

**Affiliations:** 1 Department of Paediatrics, Division of Oncology/Haematology, VU University Medical Center (VUmc), De Boelelaan 1118, 1081 HZ Amsterdam, The Netherlands; 2 Department of Biostatistics and Epidemiology, Cincinnati Children’s Hospital Medical Center, 3333 Burnet Avenue, Cincinnati, OH 45229 USA; 3 Department of Pediatrics, Cancer and Blood Diseases Institute, Cincinnati Children’s Hospital Medical Center, 3333 Burnet Avenue, Cincinnati, OH 45229 USA; 4 Department of Epidemiology and Biostatistics, VU University Medical Center (VUmc), De Boelelaan 1089a, 1081 HV Amsterdam, The Netherlands; 5 Department of Radiology & Nuclear Medicine, VU University Medical Center (VUmc), De Boelelaan 1118, 1081 HZ Amsterdam, The Netherlands; 6 Department of Neurosurgery, Neurosurgical Center Amsterdam, Room 2F 020, De Boelelaan 1117, 1081 HV Amsterdam, The Netherlands; 7 Academy of Princess Máxima Center for Pediatric Oncology, Postbus 85090, 3508 AB Utrecht, The Netherlands; 8 Department of Radiology, Cincinnati Children’s Hospital Medical Center, 3333 Burnet Avenue, Cincinnati, OH 45229 USA; 9 Department of Paediatric Oncology/Haematology, VU University Medical Center, De Boelelaan 1118, Room KTC4.027, 1081 HZ Amsterdam, The Netherlands

**Keywords:** Prognostic modeling, Cox proportional hazards modeling, External validation, Discrimination, Calibration

## Abstract

We aimed to perform external validation of the recently developed survival prediction model for diffuse intrinsic pontine glioma (DIPG), and discuss its utility. The DIPG survival prediction model was developed in a cohort of patients from the Netherlands, United Kingdom and Germany, registered in the SIOPE DIPG Registry, and includes age <3 years, longer symptom duration and receipt of chemotherapy as favorable predictors, and presence of ring-enhancement on MRI as unfavorable predictor. Model performance was evaluated by analyzing the discrimination and calibration abilities. External validation was performed using an unselected cohort from the International DIPG Registry, including patients from United States, Canada, Australia and New Zealand. Basic comparison with the results of the original study was performed using descriptive statistics, and univariate- and multivariable regression analyses in the validation cohort. External validation was assessed following a variety of analyses described previously. Baseline patient characteristics and results from the regression analyses were largely comparable. Kaplan–Meier curves of the validation cohort reproduced separated groups of standard (n = 39), intermediate (n = 125), and high-risk (n = 78) patients. This discriminative ability was confirmed by similar values for the hazard ratios across these risk groups. The calibration curve in the validation cohort showed a symmetric underestimation of the predicted survival probabilities. In this external validation study, we demonstrate that the DIPG survival prediction model has acceptable cross-cohort calibration and is able to discriminate patients with short, average, and increased survival. We discuss how this clinico-radiological model may serve a useful role in current clinical practice.

**Electronic supplementary material:**

The online version of this article (doi:10.1007/s11060-017-2514-9) contains supplementary material, which is available to authorized users.

## Introduction

Diffuse intrinsic pontine glioma (DIPG) is a highly aggressive tumor in the pons that nearly exclusively affects children. Prognosis is dismal, with a median overall survival (OS) of 9 months [1]. Despite decades of research, survival has not improved, although variations in outcome have been reported [2]. Given the rarity of DIPG, clinical trials are mostly non-randomized and include low patient numbers. Results are, therefore, possibly influenced by selection bias since prognostic variables for patient stratification are rarely taken into account. This makes it difficult to determine whether the observed variations in survival are caused by true treatment effects or (patient- or disease-related) confounders [3]. At the same time, by not taking into account significant prognostic variables in small-scaled clinical trial cohorts, the detection of potential subgroup-specific efficacy may be hampered [4].

To better understand the variables influencing the outcome of DIPG patients, a multivariable prediction model was developed to assess survival, based on radiographic and clinical variables [5]. For this, patient data from the Netherlands, United Kingdom and Germany, now included in the SIOPE DIPG Registry [6], were used. The DIPG survival prediction model that was developed contained four prognostic variables, including patient age and symptom duration at time of diagnosis, presence of ring enhancement on diagnostic MR-imaging, and receipt of any chemotherapy at any time during the disease course, and could distinguish patients with short, average, and increased survival. Internal validation of the model by bootstrapping of the original dataset showed acceptable calibration. External validation, however, could not be performed because a large-scale independent dataset was lacking. With the recently established close international collaboration between the SIOPE DIPG Registry and International DIPG Registry [6, 7], such dataset became available.

The primary aim of this study was therefore to perform external validation of the DIPG survival prediction model, using an independent and unselected cohort of patients from the International DIPG Registry [7]. External validation is essential to determine a model’s accuracy and examine its generalizability [8–10]. An accurate and generalizable model not only discriminates well between patient outcomes, thereby dividing the cohort into distinguishable risk groups, but also calibrates well to prevent under- or over prediction of the survival probabilities. A secondary aim of this study was to discuss the utility of the current clinico-radiological DIPG survival prediction model, considering the rapid developments in the field of DIPG research, especially the discovery of biological variables that correlate with survival.

## Materials and methods

### Study population

For external validation of the DIPG survival prediction model, an independent and unselected cohort from the International DIPG Registry was utilized. This cohort contained comparable data to the original cohort. However, data collected differed based on participating countries and sites, time frame of data collection, and participating investigators and coordinators responsible for data collection. The same inclusion criteria were used for patients registered on both the International DIPG Registry and the SIOPE DIPG Registry; the common definition of DIPG included a T1-weighted hypointense and T2-weighted hyperintense tumor with at least 50% involvement of the pons [6, 7, 11]. As described previously, all patients had central review of diagnostic MR-images by two board certified neuroradiologists (JL and BVJ) to confirm the diagnosis DIPG [7]. As in the original study, only patients aged 0–18 years were included.

The derivation cohort used in the original study included patients diagnosed between 1990 and 2010. In that study, data were abstracted from two nationwide cohorts (the Netherlands and Germany) and one single center cohort (United Kingdom) [5]. The validation cohort consisted of patients diagnosed between 1999 and 2015. Patients from the United States, Canada, Australia and New Zealand were enrolled in the International DIPG Registry through the website, dipgregistry.org, or via collaborating medical centers. Data for this study were abstracted by registry staff.

### DIPG survival prediction model

The prognostic variables in the DIPG survival prediction model were age (age ≥3 years = 1/age <3 years = 0) and symptom duration at time of diagnosis (as continuous variable), presence of ring enhancement on diagnostic MR-imaging (yes = 1/no = 0), and the use of oral or intensive (i.v.) chemotherapy at any time during the disease course (yes = 1/no = 0). This model was converted into the following clinical prediction rule: (age ≥3 years × 7) + (symptom duration in months × −1) + (ring enhancement × 4) + (oral chemotherapy and/or intensive chemotherapy × −4). With the resulting risk score, the predicted risk of death at 12 months can be calculated for each individual DIPG patient to whom the rule is applied. In the original study, patients with a risk-score <1 were considered to represent a standard risk group and showed a median survival of 13.7 (+1.7) months, patients with a risk-score of 1–6 were considered to represent an intermediate risk group with a median survival 9.7 (+0.4) months, and patients with a risk-score >6 were considered to represent a high-risk group with a median survival of 7.0 (+0.9) months.

### Variables

The variables as used in the original study were retrieved from the International Registry. For most variables, the exact same definition and scoring system were used. One radiographic variable, encasement of the basilar artery, was not collected in the validation cohort, but this was not a significant predictor of prognosis in the original study. Also, due to the similarity of the hazard ratios for oral and intensive chemotherapy, these variables were combined to form the variable “any chemotherapy” for the prediction rule in the original study. For this reason, we have considered only “any chemotherapy” in this validation study, defined as the receipt of chemotherapeutics at any time during the disease course. For histology, the 2007 WHO grading system [12] was used in both cohorts, however, tissue was collected at different time points during the disease course: in the derivation cohort from biopsy alone, and in the validation cohort from biopsy and autopsy. The outcome variables collected were the event (e.g. death) and time until the event (e.g. OS). OS was defined as the time from diagnosis to death.

### Missing data

Multivariable analyses and external validation steps were performed using the complete cases, single and multiple imputation from the validation cohort [13, 14]. The complete cases were patients with complete data on the four prognostic and two outcome variables.

### Data analyses

All analyses were performed using data from the validation cohort only. The results from the analysis in the validation cohort were compared to the original results previously published [5]. Continuous and categorical patient characteristics were summarized by median (range) and frequency (percent), respectively, to enable basic comparison with the results from the original study [5]. Univariate and multivariable hazard ratios were found using Cox proportional hazards regression analysis for all variables of interest, and compared to the Hazard ratios found in the original study [5]. To externally validate the DIPG survival prediction model, the methods as described by Royston et al. were performed [15]. Statistical significance was assessed at the 0.05 level.

#### Method 1: regression on the Prognostic Index

The Prognostic Index (PI) is the weighted sum of the prognostic variables, where the weights are the regression coefficients from the derivation cohort A Cox proportional hazards model was fit with the PI as the only prognostic variable. A calibration slope smaller than 1 indicates suboptimal discrimination. A score test was performed to test for if the slope was significantly different from 1 Averaged values were reported as a result of multiple imputations.

#### Method 2: model misspecification/fit

Model fit was defined as the agreement of the regression coefficients between the derivation and validation cohorts. It was assessed by fitting a Cox model that included the prognostic variables and the PI (using the original coefficients from the derivation cohort) as an ‘offset’ variable. The model is considered to fit well if the regression coefficients for the prognostic variables were not statistically significantly different from 0. This was tested jointly for significance using a pooled likelihood ratio (LR) test from each multiple imputation.

#### Method 3: measures of discrimination

To determine the discriminative ability of the DIPG survival prediction model, the Harrell’s c-index of concordance was calculated in the validation cohort. Harrell’s c-index reflects the proportion of all patient pairs in which the predicted and observed outcomes are accordant [16]. An index value close to 1 is considered to reflect good performance of the model. Results were pooled over multiple imputed datasets by taking the average.

#### Method 4: Kaplan–Meier curve for risk groups

Kaplan–Meier curves for OS were created based on the three risk groups from the original study, including standard risk (score <1), intermediate risk (score 1–6), and high-risk (score >6) groups. The Kaplan–Meier curves allowed a visual evaluation of the discriminative ability of the DIPG survival prediction model when applied to the data from the validation cohort. The Kaplan–Meier curves also indicated how well the model is calibrated by means of comparing agreement of the curves from the derivation and validation cohorts.

#### Method 5: hazard ratios across risk groups

To check the discriminative ability represented by the Kaplan–Meier curves, hazard ratios across the risk groups were calculated. Ideally, each value would correspond well with what was observed in the results from the original study.

#### Method 6: probability of death

The calibration of the DIPG survival prediction model in the validation cohort was also checked by using a calibration curve. On this curve the predicted and observed probabilities to die at 12 months were plotted. The baseline survival probability for 12 months’ survival in the validation cohort was determined using [S_0_(12)]. The survival probabilities at 12 months were calculated using S(12) = S_0_(12)^exp(PI)^, where S_0_(12) = 0.39506 and the probability of dying at 12 months was 1 − S(12).The results were compared with the results in the original study.

### Statistical software

Data cleaning and statistical evaluation was carried out using R (Vienna, Austria, R foundation for Statistical Computing, Version 3.1.3). Multiple imputation (MI) and single imputation (SI) were performed by use of the mi package [17]. For MI, total of 100 imputations was used.

## Results

### Derivation and validation cohorts

The derivation cohort comprised 316 typical DIPG patients [5]. The validation dataset includes 249 patients (Table 1). Following the inclusion criteria from the original study, patients >18 years of age and patients with non-typical pontine tumors, based on the classification criteria of Barkovich et al. [11] were excluded. Out of 249 patients, 205 are considered complete cases based on the prognostic and outcome variables from the DIPG survival prediction model. Out of 249 patients, seven patients had missing values in at least one of the outcome variables. In the remaining 242 patients, missing values for the prognostic variables were substituted by single and multiple imputation methods. These datasets were used for the multivariable analyses and external validation steps. Results from the multiple imputation methods are discussed below, results from complete case and single imputation methods can be found in the Supplementary material.

**Table 1:** Baseline characteristics of children with a diffuse intrinsic pontine glioma

Category	Baseline variable	Derivation^a^	Validation
		n (%)	n (%)
Total		316	249
Sex	Female	156 (51)	137 (55)
	Male	160 (49)	110 (45)
Age	Mean age [years (range)]	7.2 (0–18)	7.1 (0.2–18.2)
	Age <3 years	20 (6)	16 (7)
	*Missing*	*–*	*3*
Signs and symptoms	Mean symptom duration pre-diagnosis, mo (range)	2.0 (0–30)	1.4 (0–12)
	Symptom duration ≥6 months	21/285 (7)	7/230 (3)
	Symptom duration <6 months	264/285 (93)	223/230 (97)
	Cranial nerve palsy	226/310 (72)	130/206 (63)
	Ataxia	192/315 (61)	127/208 (61)
	Pyramidal tract symptoms	133/317 (42)	84/210 (40)
Histology	WHO II	14/68 (21)	10/57 (18)
	WHO III	21/68 (31)	15/57 (26)
	WHO IV	26/68 (38)	28/57 (49)
	High-grade glioma not defined	7/68 (10)	4/57 (7)
	Unknown (no biopsy or biopsy/autopsy)^b^	248/316 (79)	192/249 (77)
MRI characteristics	Pontine involvement 50–66%	33/316 (10)	9/249 (4)
	>67%	283/316 (90)	240/249 (96)
	Ring enhancement	114/316 (36)	73/235 (31)
	No contrast given	14/316 (4)	Not collected
	Encasement basilar artery		Not collected
	180° < encasement < 360°	212/316 (67)	*–*
	Full encasement (360°)	71/316 (23)	*–*
	No encasement	33/316 (10)	*–*
	Hydrocephalus	65/316 (21)	57/228 (25)
	Growth in mesencephalon	183/316 (58)	174/249 (70)
	Growth in medulla oblongata	124/316 (39)	186/249 (75)
Treatment	Radiotherapy	272/299 (91)	234/241 (97)
	Oral chemotherapy^c^	159/316 (50)	*–*
	Intravenous chemotherapy^d^	33/316 (10)	*–*
	Any chemo	–	182/236 (77)
Outcome	Median overall survival (OS), mo	10 (±0.38)	10.7 (±0.35)
	12-month OS	35%	40%
	24-month OS	9%	8%
	5-year OS	2%	0%
	Median PFS, mo	6 (±0.25)	6 (±0.5)

^a^Data directly copied from the original study [5]

^b^In the derivation cohort, tissue was collected from biopsy (n = 68). In the validation cohort, tissue was collected from biopsy and autopsy (n = 57)

^c^Patients were mainly treated with temozolomide concurrent with and/or adjuvant to radiotherapy or with vincristine and carboplatin according to the SIOP LGG protocol

^d^ *HITGBM-D* pre-irradiation methotrexate, radiation and cisplatin, etoposide, vincristine and ifosfamide, *HITSKK* cyclofosfamide, methotrexate and vincristine or DIPG-VUMC-1 containing high dose chemotherapy with stem cell reinfusion

### Comparison of the study populations

Table 1 presents the patient characteristics of both the validation cohort and the derivation cohort (copied from the original paper). The distribution of most variables within the cohorts is remarkably similar, however, small differences are seen in the prevalence of cranial nerve palsies between the derivation and the validation cohort (72 vs. 63%, respectively). Also, the validation cohort shows a shorter duration of symptoms pre-diagnosis (max 12 vs. 30 months), an 11% higher prevalence of WHO grade IV histology, a higher prevalence of tumors that affect >67% of the pons (96 vs. 90%) and a higher prevalence of tumors that extend towards the mesencephalon and medulla oblongata (12 and 36% higher, respectively). The validation cohort also contains a higher percentage of patients who have been treated with either radiotherapy (97 vs. 91%) and/or chemotherapy at any time during the disease course (77 vs. 60%). The 5-years’ OS of the validation cohort was 0% (vs. 2% in the derivation cohort), however, with a median OS of 10.7 (±0.35) versus 10 (±0.38).

Table 2 shows the comparison of hazard ratios for each variable investigated in the original study, resulting from univariate analysis. The variables included in the DIPG survival prediction model are indicated with an arrow. The hazard ratios for these variables, i.e. age ≥3 years, symptom duration, presence of ring enhancement, and chemotherapy, point in the same direction in both cohorts. In the validation cohort, significance is only found for the use of chemotherapy.

**Table 2:** Results of the univariate Cox proportional hazards regression analysis for the variables of interest

	Baseline variables	Hazard ratios (95% CI) and p values			
		Derivation^a^		Validation	
	Increasing age (years)	1.01 (0.98–1.04)	0.68	0.97 (0.93–1.00)	0.034
→	Age ≥3 years	2.19 (1.25–3.82)	0.006	1.28 (0.75–2.19)	0.370
	Sex (male vs. female)	0.92 (0.72–1.17)	0.49	1.07 (0.83–1.37)	0.63
	Signs and symptoms				
→	Symptom duration (months)	0.90 (0.86–0.95)	0.0001	0.93 (0.86–1.01)	0.074
	Cranial nerve palsy	1.29 (0.97–1.70)	0.08	1.22 (0.91–1.64)	0.170
	Pyramidal tract symptoms	1.18 (0.93–1.50)	0.17	1.00 (0.75–1.32)	0.990
	Ataxia	1.38 (1.07–1.79)	0.02	0.86 (0.65–1.15)	0.310
	MRI characteristics				
	Pontine involvement: 33/50–67% vs. >67%	1.29 (0.86–1.92)	0.21	1.14 (0.59–2.23)	0.69
→	Ring enhancement	1.53 (1.19–1.97)	0.001	1.18 (0.90–1.57)	0.23
	Encasement basilar artery				
	>180°; <360° vs. no encasement	1.15 (0.77–1.73)	0.49	–	–
	360° vs. no encasement	1.30 (0.83–2.05)		–	–
	Hydrocephalus	0.95 (0.71–1.28)	0.75	1.31 (0.97–1.78)	0.080
	Growth in mesencephalon	0.93 (0.73–1.18)	0.54	1.02 (0.78–1.35)	0.860
	Growth in medulla oblongata	1.17 (0.92–1.48)	0.22	1.21 (0.91–1.63)	0.190
	Histology				
	WHO grade III–IV vs. grade II	1.55 (0.80–3.00)	0.20	1.57 (0.81–30.06)	0.180
	Treatment				
→	RT and chemotherapy vs. RT	–	0.004	–	–
	Oral chemotherapy	0.64 (0.49–0.84)	–	–	–
	Intravenous chemotherapy	0.68 (0.45–1.02)	–	–	–
	Any chemotherapy	–	–	0.48 (0.35–0.66)	<0.0001

*CI* confidence interval, *RT* radiotherapy, → prognostic variable included in the DIPG survival prediction model

^a^Data directly copied from the original study [5]

Table 3 shows the comparison of hazard ratios resulting from multivariable analyses. Again, all predictor variables point in the same direction, but in the validation cohort significance is only found for the use of chemotherapy.

**Table 3:** Results of the multivariable Cox proportional hazards regression analysis for the prognostic variables

Predictor	Hazard ratios (95% CI) and p values			
	Derivation^a^		Validation^b^	
Age ≥3 years	1.95 (1.01–3.80)	0.046	1.29 (0.72–1.84)	0.38
Symptom duration (months)	0.92 (0.86–0.97)	0.003	0.93 (0.85–1.01)	0.11
Ring enhancement	1.41(1.07–1.84)	0.013	1.07 (0.78–1.36)	0.63
RT and chemotherapy vs. RT	0.65 (0.49–0.99)	0.013	0.51 (0.20–0.82)	< 0.0001

^a^Data directly copied from the original study [5]

^b^Results from multiple imputation method analyses (n = 242, 7 patients were missing survival time and/or event status)

### External validation steps

#### Method 1: regression on the Prognostic Index

The slope in the Cox proportional hazards model on the PI in the validation cohort was 0.72 and different from 1 (p = 0.01). This suggests a suboptimal discrimination and some mis-calibration of the model.

#### Method 2: model misspecification/fit

The agreement, or rather the above suggested difference (i.e. a slope of 0.72), in one or more regression coefficients between the derivation and validation cohort was tested by creating and ‘offset’ Cox proportional hazards model. The joint test of all the prognostic variables resulted in a chi^2^ of 9.77, which was different from 0 (p = 0.002), suggesting not a good fit of the PI in the validation cohort.

#### Method 3: measures of discrimination

Harrell’s c-index in the original study was 0.68 Harrell’s c-index was 0.58 in the validation cohort, which reflects modest discrimination, i.e. good separation between survival curves for individuals or groups.

#### Method 4: Kaplan–Meier curves for risk groups

**Fig. 1:**
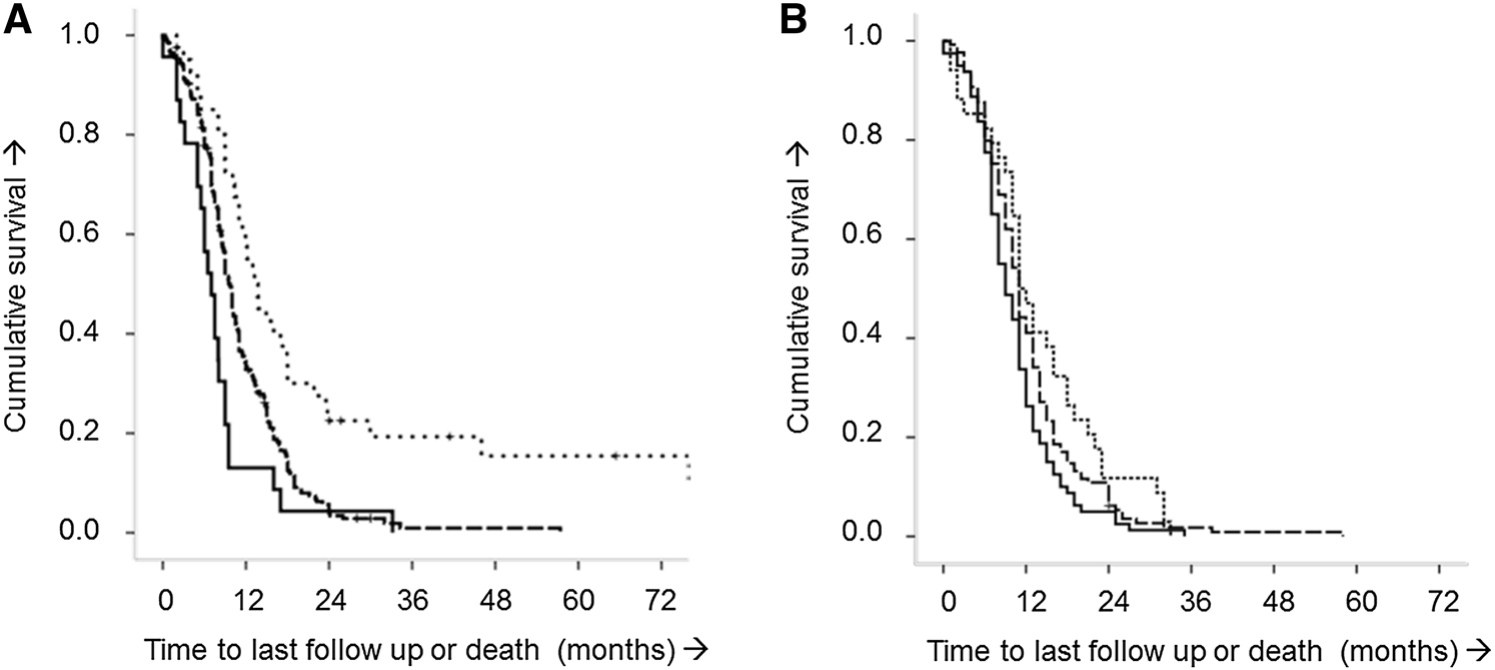
Kaplan–Meier curves presenting the risk groups in the derivation (**a**) and validation (**b**) cohort. **a** Derivation cohort (data directly copied from the original study [5]). *Dotted lines* Risk score <1: standard risk group. *Dashed lines* Risk score 1–6: intermediate risk group. *Solid lines* Risk score ≥7: high-risk group. **b** Validation cohort. *Dotted lines* Risk score <1: standard risk group (n = 39). *Dashed lines* Risk score 1–6: intermediate risk group (n = 125). *Solid lines* Risk score ≥7: high-risk group (n = 78)

Figure 1 displays the Kaplan–Meier curves for both the derivation cohort (A) and the validation cohort (B) when preserving the three risk groups from the DIPG survival prediction model. Both KM-curves show separated lines, thereby dividing the cohort in three distinguishable risk groups. In both cohorts, the KM-curves show that a patient in the standard risk group has approximately two times greater odds of surviving past one year than a patient in the high-risk group. When comparing the individual curves in the validation and derivation cohorts, however, these do not seem to match perfectly. Especially the standard risk group in the validation cohort does not separate as well in the first 9 months after diagnosis as in the derivation cohort.

#### Method 5: hazard ratios across risk groups

Table 4 presents the hazard ratios across the risk groups. The hazard ratios are well maintained in the validation cohort (i.e. they point in the same direction as in the original study) and are significantly different between risk groups. The results also reflect the Kaplan–Meier curves: the more widely separated lines (representing the standard vs. high-risk group) have a larger hazard ratio. This again confirms that the model is able to discriminate between patients with short, average and increased OS.

**Table 4:** Hazard ratios across the risk groups

	Hazard ratios (95% CI) and p values^a^					
	Intermediate vs. standard		High vs. standard		High vs. intermediate	
Multiple Imputation averaged risks^b^	1.29 (0.9–1.68)	0.20	1.67 (1.26–2.08)	0.014	1.29 (1.00–1.58)	0.09

^a^Results from multiple imputation method analyses (n = 242, 7 patients were missing survival time and/or event status)

^b^This finds the risk group most often assigned from all imputations

#### Method 6: probability of death

The calibration curve, presented in the Supplementary material, shows that the predicted probabilities to die within 12 months in the validation cohort are underestimated. All closed circles are above the line (i.e. symmetric), suggesting this to be dependent upon the baseline survival function.

## Discussion

External validation of a Cox prediction model is seldom described in the literature although it is an essential step towards acceptance of a model into clinical practice [2]. Unvalidated models should not be used in clinical practice [9]. Since DIPG is a rare orphan disease, external validation using a large-scale independent dataset is extremely challenging. This study describes the external validation of the previously published DIPG survival prediction model [5] in an independent and unselected cohort from the International DIPG Registry, including DIPG patients from the United States, Canada, Australia and New Zealand. It is the first study resulting from the recently established close international collaboration between the SIOPE DIPG Registry and International DIPG Registry [6, 7]. This study represents the welcome paradigm shift in DIPG research, in which data are no longer a rate-limiting resource.

The results of this external validation study confirm that the DIPG survival prediction model, which combines three favorable predictors (age <3 years and longer duration of symptom at time of diagnosis and use of oral or intensive chemotherapy at any time during the disease course) and one unfavorable predictor (presence of ring enhancement on diagnostic MR-imaging) is able to reproduce separated groups of standard, intermediate, and high-risk patients.

For the statistical approach of external validation, Royston et al. provided well worked-out methods to determine the discriminative and calibration abilities of a survival prediction model. For survival prediction modeling in particular, discrimination is the key indicator of model accuracy because this reflects its capacity to separate individual patient outcomes into distinguishable risk groups. In our validation cohort, the slope of the PI, Harrell’s c-index and Kaplan–Meier curves suggested poorer discrimination, but this is well within the range of what may generally be expected in validation studies [9]. Notably, the hazard ratios across the risk groups seen in the derivation cohort are well maintained in the validation cohort. Although not statistically significant, which is also generally expected in external validation studies, the hazard ratios all point in the same direction as in the original study. This is confirmed by the Kaplan–Meier curves that show separation of the lines for each risk-group. Overall, the results from this external validation study suggest *adequate* discriminative and calibration abilities of the DIPG survival prediction model. While most prognostic models have a poorer performance in new datasets, the performance of our model remained stable over datasets [9]. We, therefore, conclude that this external validation is succesful, meaning the model has acceptable performance and that it is generalizable in DIPG patients. However, this finding does not imply that the model itself, is perfect.

Finding a slightly lower discriminative ability in external validation studies is not surprising, and generally due to (i) overestimation of the model in the derivation cohort. This is most likely the case in this external validation study, since internal validation of the model by bootstrapping revealed a 15% overfit in the original study [5]. Discrepancy in discriminative ability may also arise when (ii) regression coefficient(s) of variables differ from the original study. This may be caused by inter-observer variation or variation in the definition of variables, or methods of measurement. It is therefore important to consider the comparability of the patients and settings. In this external validation study, an example would be the observed difference in prevalence of WHO grade IV histology, which is caused by the fact that in the derivation cohort tissue was collected from biopsy alone, while in the validation cohort tissue was collected both from biopsy and autopsy. It should also be noted that “any chemotherapy” reflects many different treatment regimens, which are not further analyzed but which are known to differ between the derivation and validation cohorts. All other variables analyzed in this study, however, were considered to be uniform to the original study variables, since both registries make use of collaboratively developed, comparable, standardized Case Report Forms (CRFs) for all variables [6, 7]. Finally, finding lower discriminative ability may also be due to (iii) case mix, meaning a “true” difference in the underlying population. Case mix in this study may be expressed in the observed shorter duration of symptoms pre-diagnosis and larger tumors, which more frequently extended towards surrounding brain structures in the validation cohort, suggesting these patients to be more affected. The assumed difference in baseline survival function, to the prejudice of the validation cohort, is underlined by the calibration curve that shows a symmetric underestimation of the predicted probabilities to die within 12 months for the latter population. It may also explain why the number of patients who received treatment was higher in the validation cohort (6% higher for radiotherapy and 17% higher for chemotherapy). Unfortunately, we could not perform additional analysis to identify possible underlying biological variations that could explain these differences between the cohorts. Due to the retrospective nature of this study, biological data on the recently discovered histone mutational status was missing for a high number of patients.

Univariate and multivariate analyses, surprisingly, showed no significant correlation between three of the predictors and overall survival in this validation cohort, while in the derivation cohort [5] and in previously published studies age [18], symptom duration [19] and ring enhancement [20] were significantly associated with prognosis. The lack of statistical significance noted in correlations on the univariate and multivariate analyses may be due to the fact that this external validation is slightly underpowered. Other factors, including the above described overestimation of the model, variation in the use of variables or case mix are also possible.

Overall, for both the development and validation of the DIPG survival prediction model, a possible limitation could be the use of disease registry data. Registries in general harbor enrollment bias with tendency for patients with unique characteristics, which in this case is mainly based on the participating institutions (with a tendency for large academic centers), and patients who self-refer. The registries, however, both aim to include *all* patients diagnosed with DIPG, both in- and outside clinical trials and both those who do or do not undergo treatment. A major strength of this study compared to other published reports on DIPG, was the requirement for central radiological review of diagnostic imaging by specialized pediatric neuro-radiologists. All patients included in the study are “typical” DIPG patients, based on the generally accepted definition of Barkovich et al. [11]. It may, therefore, be expected that the SIOPE and International DIPG Registry contain comparable data that are representative for the “general” DIPG population. In a rare orphan disease such as DIPG, where the lack of large-scale dataset for decades has been the rate-limiting resource, we consider this study a valuable step forward.

Having a reliable and applicable model to predict the survival in DIPG patients is of great clinical relevance [21]. As discussed, results of clinical trials are possibly influenced by selection bias since prognostic variables are rarely taken into account and trials are largely underpowered. The survival prediction model will be particularly useful for stratification of patients by disease severity before they enroll on clinical trials, or for interpretation of treatment outcomes based on risk stratification. Stratification is important to determine whether an observed change in survival can be attributed to the novel therapeutic intervention or, alternatively, to selection bias. Intriguingly, both the original study, as well as the validation study, showed significant survival benefit for patients who received chemotherapy, in contrast to the disappointing results of individual studies investigating the use of chemotherapy in DIPG patients [1]. It would therefore be interesting to apply the DIPG survival prediction model to all historical trial data from the literature, in which such DIPG risk stratification has not been taken into account. It is possible that the identification of effective therapies has been hampered by selection of solely high-risk patients resulting in false-negative results, and, vice versa, more favorable (‘false-positive’) results by selection of relatively more standard-risk patients. By retrospectively applying the DIPG survival prediction model, beneficial or negative effects of certain treatment strategies may still be identified. The recently developed infrastructure of both the SIOPE and International DIPG Registry [6, 7], including central radiology review of DIPG patients, provides the opportunity to perform such a study, as a total of over 1400 patients (of whom many participated in clinical trials) have now been enrolled on both Registries.

Currently, the DIPG survival prediction model does not include biological variables. Castel et al. recently showed that type of histone H3 mutation is a strong prognostic variable of survival [22]. Based on the recent discoveries, our clinico-radiologically-defined risk groups are likely based on underlying biological variations [4]. However, since biopsies are still not routinely performed in the world, for most patients, tumor material for mutational status analyses is not (yet) readily available [3]. Due to a lack of biological data, the current study was not aimed at updating the DIPG survival prediction model to also including biological variables. In fact, until biopsies are routinely performed, a model including biological variables would not yet be generalizable. We emphasize the value of the discovery of biological variables, but underline the current clinical utility and versatility of this clinico-radiological model to easily stratify DIPG patients without extensive biological analysis [23]. In the future, when biopsies become standard of care, the incorporation of biological variables may further improve the DIPG survival prediction model, but until that time, this clinico-radiological model may perform a useful role in risk-classification of DIPG patients.

## Electronic supplementary material

Below is the link to the electronic supplementary material.

Supplementary material 1 (DOCX 58 KB)

Supplementary material 2 (DOCX 63 KB)
