# Supplementary material for: External validation of the diffuse intrinsic pontine glioma survival prediction model: a collaborative report from the International DIPG Registry and the SIOPE DIPG Registry
Source: J Neurooncol. 2017 May 30;134(1):231–40. doi: 10.1007/s11060-017-2514-9 (PMC5543206; doi:10.1007/s11060-017-2514-9)
Supplement: Supplementary file 1 — Supplementary material 1 (DOCX 58 KB) [file 11060_2017_2514_MOESM1_ESM.docx]

**Supplementary material 1. Results from complete case and single imputation methods**

Multivariable Cox proportional hazards regression analysis for the prognostic variables

|  | **Hazard Ratios and p-values** | | | | | |
| --- | --- | --- | --- | --- | --- | --- |
| **Predictor** | **Derivation***  (n=316) | **p-value*** | **Validation Complete Cases** (n=205) | **p-value** | **Validation Single Imputation** (n=242)** | **p-value** |
| Age ≥ 3 years | 1.95 | 0.046 | 1.02 | 0.94 | 1.21 | 0.49 |
| Increasing symptom duration (months) | 0.92 | 0.003 | 0.94 | 0.17 | 0.94 | 0.15 |
| Ring enhancement | 1.41 | 0.013 | 1.08 | 0.64 | 1.08 | 0.25 |
| RT & chemotherapy versus RT: | 0.65 | 0.013 | 0.52 | <0.0001 | 0.47 | <0.0001 |

** Data directly copied from the original study [5].*

****** *n=242, 7 patients were missing survival time and/or event status*

Method 1: Regression on the Prognostic Index

|  | **Slope of PI** | **p-value** |
| --- | --- | --- |
| Complete cases (n=205) | 0.66 | 0.0024 |
| Single Imputation (n=242)* | 0.75 | 0.0002 |
| Multiple Imputation (n=242)* | 0.72 | 0.0005 |

***** *n=242, 7 patients were missing survival time and/or event status*

Method 2: Model misspecification/fit

|  | **Chi^2^** | **p-value** |
| --- | --- | --- |
| Complete cases (n=205) | 10.98 | 0.027 |
| Single Imputation (n=242)* | 12.33 | 0.015 |
| Multiple Imputation (n=242)* | 9.77 | 0.002 |

***** *n=242, 7 patients were missing survival time and/or event status*

Method 3: Measures of discrimination

|  | **Harrell's c-index** | **Standard error** |
| --- | --- | --- |
| Complete cases (n=205) | 0.57 | 0.490 |
| Single Imputation (n=242)* | 0.58 | 0.044 |
| Multiple Imputation (n=242)* | 0.58 | 0.044 |

***** *n=242, 7 patients were missing survival time and/or event status*

Method 5: Hazard ratios across risk groups

|  | **Hazard ratios** | | |
| --- | --- | --- | --- |
| **Risk groups** | **Intermediate vs. Standard** | **High vs. Standard** | **High vs. Intermediate** |
| Complete cases (n=205) | 1.23 | 1.57 | 1.27 |
| Single Imputation (n=242)* | 1.20 | 1.69 | 1.41 |
| Multiple Imputation (n=242)* averaged risks** | 1.27 | 1.63 | 1.28 |

***** *n=242, 7 patients were missing survival time and/or event status*

*** This finds the risk group most often assigned from all imputations*
