# Supplementary material for: External validation of the diffuse intrinsic pontine glioma survival prediction model: a collaborative report from the International DIPG Registry and the SIOPE DIPG Registry
Source: J Neurooncol. 2017 May 30;134(1):231–40. doi: 10.1007/s11060-017-2514-9 (PMC5543206; doi:10.1007/s11060-017-2514-9)
Supplement: Supplementary file 2 — Supplementary material 2 (DOCX 63 KB) [file 11060_2017_2514_MOESM2_ESM.docx]

**Supplementary material 2. Figure displaying the observed and predicted probability of death**


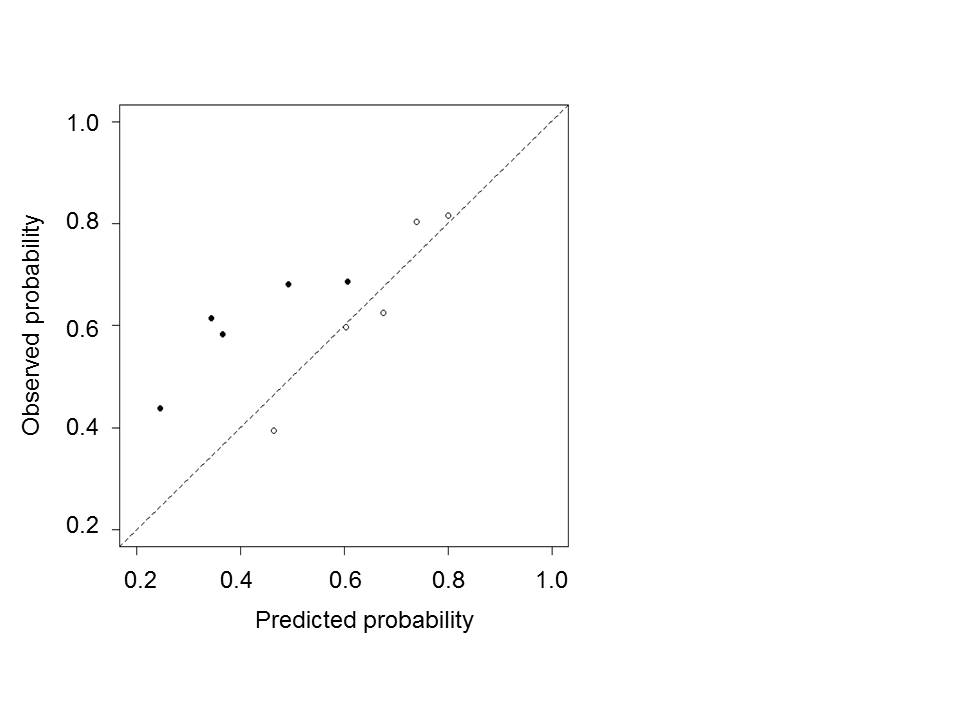


Open circles – Derivation cohort

Closed circles – Validation cohort (complete cases)
